# The Relationship between Muscle Ultrasound Parameters and Diabetic Peripheral Neuropathy among Patients with Type 2 Diabetes Mellitus: A Cross-Sectional Study

**DOI:** 10.1155/2023/8897065

**Published:** 2023-12-19

**Authors:** Yanling Zhong, Xiaojia Liu, Teng Lin

**Affiliations:** ^1^Department of Ultrasound, The First Affiliated Hospital of Shantou University Medical College, Shantou, Guangdong 515000, China; ^2^Shantou University Medical College, Shantou, Guangdong 515000, China

## Abstract

**Background:**

Muscle dysfunction is an early complication of diabetic peripheral neuropathy (DPN). As a convenient and low-cost tool, muscle ultrasound has been used to assess muscle quality and muscle mass. However, the relationship between different muscle ultrasound parameters and DPN is unclear.

**Objectives:**

This study was designed to investigate the relationship between ultrasound parameters of different muscles and DPN among patients with type 2 diabetes mellitus, including the rectus femoris (RF), tibialis anterior (TA), and medial head of gastrocnemius (MG).

**Materials and Methods:**

The research enrolled 90 patients with type 2 diabetes mellitus (T2DM). All images were attained from both sides. Muscle measurements contained muscle thickness (MT), cross-sectional area (CSA), echo intensity (EI), and corrected EI. The binary logistic regression and multiple linear regression were used to investigate the association between muscle ultrasound parameters and DPN or vibration perception threshold (VPT).

**Results:**

EI, corrected EI, MT of MG, and EI of TA were associated with DPN separately after adjusting other clinical variates. Among these muscle parameters, the EI of MG had a better predictive value (OR: 1.114, 95% CI: 1.039, 1.196) of DPN. Combined with CSA of RF, peripheral artery disease (PAD), and sex, the corrected EI of MG was associated with the vibration perception threshold (VPT) (standard *β* = 0.242, *p* < 0.001), better than the EI of MG (standard *β* = 0.215, *p* = 0.002).

**Conclusions:**

MG (MT, EI, and corrected EI) and TA (EI) were associated with DPN, respectively. CSA of RF and corrected EI or EI of MG combined with PAD and sex were associated with VPT significantly, which supported that muscle ultrasound might be a substantial quantitative tool for detecting the exercise benefits for DPN.

## 1. Introduction

Diabetic peripheral neuropathy (DPN), one of the most common compliments of diabetic mellitus and independent risk factors of cardiovascular disease, declines patients' life quality and leads to poor outcomes, including diabetic foot and lower-extremity amputations. Present recommendations focused on foot risk detection through clinical symptoms and physical examination, combining the nerve conduction test if necessary [1]. Among the quantitative sensory testing, the vibration perception threshold (VPT) showed higher sensitivity and specificity (59.5–72.2% sensitivity and 79.8–90.2% specificity among Chinese T2DM patients) and even performed well in detecting early-stage DPN [2].

As an early landmark of DPN, muscle dysfunction concludes the decline of muscle strength, muscle mass, and changes in muscle condition. Diabetes speeds up muscular atrophy through oxidative stress and inflammation pathway [3]. A meta-analyzed research including associated cross-sectional articles based on dual-energy X-ray absorptiometry (DXA) showed that the connection between DPN and sarcopenia might be statistically significant with the pooled OR of 1.62 (95% CI: 1.30-2.02, *I*^2^ 0%) [4]. One found that increased muscle mass index predicted improvement of partial motor and sensory nerve conduction velocity among T2DM male patients in the follow-up period [5].

Unlike DXA, which assesses the whole-body muscle mass, muscle ultrasound focuses on each muscle. Muscle ultrasound showed more detailed information about muscle lately for assessing muscle mass or quality. The muscle quality was defined as the maximal muscle strength per unit of muscle size. Previous studies used muscle thickness (MT) and cross-sectional area (CSA) representing muscle size to normalize muscle strength, of which the reproducibility and reliability were reasonable compared with DXA [6]. Although there is a lack of solid evidence at the histological level, many studies have confirmed a relationship between echo intensity (EI) and muscle composition, with high EI values indicating a lower proportion of contractile components (muscle fibers) and a higher proportion of noncontractile components (fat tissue, fibrous tissue) in muscle composition [7]. It is worth noting that corrected EI might be more representative of muscle composition. Young et al. reported that EI adjusted by subcutaneous fat thickness was strongly correlated with the intramuscular fat measured by MRI, which indicated an increase in extramyocellular lipids [8]. After correcting the subcutaneous fat thickness, the relationship between EI and motor function changed simultaneously [9, 10]. Therefore, EI and corrected EI were encouraged to be reported. Since the good performance of muscle ultrasound in muscle quality, muscle ultrasound also has been suggested as an alternative screening tool for sarcopenia [11]. As an alternative tool, compared with DXA and MRI, muscular ultrasound is a quick, convenient, and low-cost tool for detecting muscle mass and muscle quality. Notably, limited by the characteristics of the ultrasound, muscular ultrasound measurement was influenced by the angle of probe orientation, image depth, and machine parameters, which highlight the need for a single assessor in the study, as well as the standardization of examination protocols.

Among various muscle groups, muscle dysfunction develops from the foot muscle to the upper part of the lower extremities. However, large muscle groups of lower extremities changed in the early DPN and influenced the gait pattern to aggravate foot muscle [12]. The rectus femoris (RF), the superficial part of the quadriceps, was focused on recently assessing the muscle mass loss and motor function of the lower extremities in older adults [13]. Low muscle mass reflected by the RF has been proven to be associated with DPN. As a pair of antagonistic muscles, the tibialis anterior (TA) and medial head of the gastrocnemius (MG) work together to keep the ankle balanced. One study observed that patients with DPN reduced adiposity and modified tissue properties of MG based on MRI measurements after a 10-week supervised training [14]. While no difference was found in MT and shear wave velocity (SWV) of TA between patients with or without DPN [15], additional studies are needed due to its essential motor function and anatomical characteristics.

Although previous articles showed that DPN is accompanied by declining muscle strength and muscle mass measured by DXA, only a few reports focused on the muscular ultrasound performance in DPN. Muscle ultrasound has been used to assess muscle mass and muscle quality among young athletes and has been applied to older people recently [16]. Studies have shown that ultrasound could detect muscle atrophy by measuring CSA, MT, and shear wave velocity (SWV) for foot muscles directly related to foot ulcers and other complications [15, 17, 18]. For large muscle groups of lower extremities, Wang et al. [19] discovered that diabetes duration over ten years and rectus femoris mass index (RFMI) lower than 2.2 cm^2^/m^2^ were indicators of DPN with an accuracy of 0.75 (95% CI: 0.72–0.79, *p* < 0.001). Zhao et al. [20] found that the muscle thickness and shear wave velocity of MG decreased among patients with DPN in the correlation analysis (*p* < 0.01). However, no direct evidence showed the echo intensity of muscle performance among T2DM patients with and without DPN. Besides, the echo intensity of the rectus femoris (RF), tibialis anterior (TA), and medial head of gastrocnemius (MG) was closely related to sarcopenia [13, 21].

Firstly, our study is aimed at investigating whether muscle ultrasound could detect early-stage changes in large muscle groups in patients with DPN and predict DPN progression. Secondly, this study also explored the differences in ultrasound parameters (CSA, MT, EI, and corrected EI) of lower extremities (including RF, MG, and TA) among patients with and without DPN.

## 2. Materials and Methods

### 2.1. Study Design

This present cross-sectional study was conducted between February 2023 and August 2023 in the First Affiliated Hospital of Shantou University Medical College. The study was approved by the ethics committee of the First Affiliated Hospital of Shantou University Medical College (approval number: B-2023-002-XZ1). Informed consent was collected from each participant before the examination. Our study enrolled 90 T2DM patients (50 males and 40 females). We recruited eligible participants from inpatients in the Department of Endocrinology who met the diagnostic criteria of type 2 diabetes mellitus according to the latest Chinese clinical guideline [22]. The exclusion criteria were as follows: (1) trauma, surgery, or other causes leading to motor dysfunction of the lower extremities; (2) myositis, dermatomyositis, fibromyositis, and other primary musculoskeletal diseases; (3) hyperthyroidism or hypothyroidism; (4) lower-extremity dysfunction due to neurological disorders; (5) decompensated cirrhosis, severe heart failure, lower-extremity arterial occlusion, uremia, and patients with cancer-accepted long-term therapy or palliative treatment; and (6) athletic patients.

### 2.2. Muscular Ultrasound Measurements

The same operator used the ultrasound device (Esaote, S.p.A, Italia, MyLab™Twice) equipped with a LA523 linear array transducer (3-12 MHz) to obtain images of muscles. The operator followed the pattern of musculoskeletal scanning but adjusted the depth and focus region based on different situations. The ultrasound measurements were taken on the rectus femoris (RF), tibialis anterior (TA), and medial head of the gastrocnemius (MG), as they had a close connection with sarcopenia and DPN [13, 21].

The operator applied ample water-soluble gel to the skin and used minimal power to obtain transverse images of each muscle vertically. Initially, the participants underwent examinations while lying on their backs. The operator received pictures of the RF at the upper one-third from the anterior superior iliac spine to the patella. Next, photos of the TA were taken from its upper part below the knee. Finally, the MG was scanned in a prone position, capturing the upper part below the popliteal space to ensure it contained the whole MG. All images were obtained on both sides of each region.

The image processing software (ImageJ, version 1.53t, National Institutes of Health, Bethesda, MD, USA) was used to measure the muscle ultrasound parameters. In the transversal images, the muscle edge, not including the muscle fascia, was depicted to circle the region of interest (ROI). The muscle thickness (MT), cross-sectional area (CSA), and echo intensity (EI) were measured in this area. The subcutaneous fat thickness was calculated from the skin surface to the muscle fascia. EI was the average grayscale value of the ROI, ranging from 0 to 255 arbitrary units, with higher values closer to white. Considering that the necessity of correcting EI for subcutaneous fat thickness is still unclear, our study assessed the corrected EI. Corrected EI = uncorrected EI + (subcutaneous fat thickness (cm) × 40.5278) [8].

### 2.3. DPN Diagnosis and VPT Measurement

The professional physician utilized the Michigan Neuropathy Screening Instrument Examination (MNSI-E) to diagnose DPN by assessing the following aspects: pinprick sensation, temperature perception, vibration perception through vibration perception threshold (VPT), pressure sensation using a 10 g monofilament, and ankle reflex. MNSI-E was recommended for clinical and epidemiological screening and assessment of DPN [1, 23]. The physician measured VPT three times at the great toes on both sides using a neurothesiometer (Beijing Laxons Technology Co., Ltd., Beijing, China). The VPT of each side was the mean value of the VPT three-time results.

### 2.4. Demographic and Other Clinical Variables

We gathered statistics on the patient's sex, age, height, weight, use of insulin, HbA1c, blood lipids (TC, TG, HDL, and LDL), peripheral artery disease (PAD) of lower extremities, diabetic kidney disease (DKD), diabetic retinopathy (DR), hypertension, smoking history, drinking history, and other medical history.

### 2.5. Statistical Analysis

The normality distribution test was confirmed by the Shapiro-Wilk test. The continuous data were expressed as mean ± SD or median (Q1-Q3), and the categorical data were shown as proportions. To compare the difference between the two groups divided by clinically diagnosed DPN, the clinical continuous data and the mean values of muscle ultrasound parameters of both lower extremities were analyzed by the nonparametric test or Student's *t*-test, and the chi-square test was used for the categorical statistics. In the binary logistic stepwise regression, only one muscle ultrasound parameter and three clinical variables (TG, TC, and PAD) were included as independent variables in each model, while the presence of DPN was the dependent variable. The Box-Tidwell test was used to prove the linear relationship between DPN and the continuous variable. Because the relationship between DPN and TG did not meet the linear relationship, TG was divided into two categories (≥2.3 mmol/L and <2.3 mmol/L). In analyzing the relationship between clinical data, muscle parameters, and VPT, each side of the lower extremity was a study object. The Spearman correlation investigated the association between continuous clinical data or muscle parameters with VPT. The relationship between the clinical categorical data (including sex, smoking, alcohol, hypertension, use of insulin, DKD, DR, and PAD of the lower extremities) and VPT was assessed by the nonparametric test. Then, multiple linear regression was applied to investigate the relationship between the associated variates and VPT. Considering the collinearity of EI and corrected EI, they were put into the regression separately. The *p* value of asymptotic significance is two-tailed, and less than 0.05 represents that the difference is statistically significant. The SPSS software (version 27.0) was utilized in the statistical analysis.

## 3. Results

Under the control of included and excluded criteria, 90 participants (39 diagnosed with DPN) accepted the assessment and examination.  Table 1 describes the essential characteristics of all participants and compares the differences between the two groups. The clinical characteristics between the two groups were similar, except that patients with DPN had a higher prevalence of PAD (*p* = 0.019) and lower levels of TC (*p* = 0.033) and TG (*p* = 0.020).  Table 2 shows the relationship between muscle ultrasound parameters and DPN in a univariate analysis. EI of the three muscles was higher in the DPN group (*p* < 0.05), and the corrected EI of TA (*p* = 0.023) and MG (*p* = 0.006) were statistically substantial. Only MT of RF and MG decreased significantly in the group with DPN. In the binary logistic stepwise regression, TC, TG, PAD, and one of the significantly important muscle measurements were included in one model. In the four models in Table 3, higher EI of MG and TA, higher corrected EI of MG, and lower MT of MG were associated with a higher prevalence of DPN. Among these muscle ultrasound parameters, the EI of MG had a better predictive value (OR: 1.114, 95% CI: 1.039~1.196). In the correlation analysis of muscle parameters, clinical variates, and VPT (Table 4), all muscle parameters were associated with VPT (*p* < 0.05), except the corrected EI of RF and TA. Among the clinical data, only TG and age made a significance.  Table 5 presents the results of multiple linear regression. Combined with sex, PAD, and CSA of RF, the corrected EI of MG was more associated with VPT (standard *β* = 0.242, *p* < 0.001) than the EI of MG (standard *β* = 0.215, *p* = 0.002). Among the clinical variates, only the prevalence of PAD (*p* < 0.001) and males (*p* = 0.036) was associated with higher VPT.

## 4. Discussion

As a complication of DPN, muscle atrophy raises the risk of falls and loss of gait balance, which has been observed in the early progress of DPN through DXA and muscle strength tests. Muscular ultrasound, a convenient quantitative assessment tool, has been used for assessing muscle mass and muscle quality in sports medicine. There was currently no evidence to support that muscle ultrasound could detect changes in proximal muscle groups during the early stages of DPN. Therefore, our study focused on and compared the morphological and histology changes of the proximal muscle of lower limbs for detecting early progression of DPN. In addition, we evaluated two-dimensional muscle ultrasound indicators for monitoring DPN progression.

In our study, although the EI of three different muscles was associated with DPN significantly, the corrected EI of RF showed no difference between the two groups, unlike TA and MG. After adjusting PAD and TG, the higher corrected EI or EI of MG, the higher EI of TA, and the lower TH of MG were independent risk factors of DPN, respectively. Although the EI and corrected EI performances still need high-level evidence, in our study, higher EI or corrected EI of MG was an independent risk factor of DPN. Previous article [15] found no difference in MT and shear wave velocity (SWV) of TA among T2DM with and without DPN. Similarly, there was no difference in MT of TA among the two groups in this study. However, the EI and corrected EI of TA were associated with DPN significantly (*p* < 0.05), and a higher EI value of TA might be the risk factor for DPN (OR: 1.086, 95% CI: 1.020~1.157). In previous studies about muscle ultrasound and motor function, EI was found to be related to muscle strength and further reflected muscle composition. As a proven indicator of motor function, EI and corrected EI might contribute to scientific guidance of exercise and training, which could be substantial indicators of supervising muscle change in DPN.

In contrast to the research conducted by Wang et al. [19], CSA of RF showed no significance among T2DM patients with and without DPN. The sample size might be the primary reason leading to the difference in the CSA of RF. Although the CSA and EI of RF were insignificant after logistic regression, the CSA of RF and EI or corrected EI of MG were associated with VPT in the multiple linear regression after adjusting PAD, sex, age, and TG. According to recent studies of diagnosing DPN, VPT decreases in the early stage of DPN. The researchers encouraged physicians to diagnose DPN through quantitative sensory testing and clinical performance because nerve conduction velocity might not change in the early stage [24]. Based on VPT performance in the early stage of DPN, this result might reflect the development of DPN. Apart from the reasons referred to, the RFMI reflected the muscle mass of the entire body, not focused on the muscle change itself. CSA of RF was often used to assess the muscle mass of the whole body in the research of sarcopenia. The inconsistency of EI and CSA of RF in Table 2 might also support that EI provided different information about muscle from other two-dimensional muscle ultrasound measurements. Moreover, males and individuals with PAD had a higher tendency to VPT compared to others, which was the same as previous research [5].

Meanwhile, we studied risk factors of DPN in T2DM patients in this research. Low muscle mass of the entire body, an underlying reason that reduces glucose uptake, promotes the progression of DPN [3]. Zhang et al. confirmed that sarcopenia, which was diagnosed through DXA, is a risk factor for DPN (OR, 1.542; 95% CI, 1.089–2.184; *p* = 0.015) and set up the nomogram for predicting DPN in a large scale research [5]. As mentioned, RFMI, an index combining RF and height to screen sarcopenia, was associated with DPN and might help predict DPN [19]. However, it was uncertain whether other muscle ultrasound measurements could predict DPN. In our study, the results of binary logistic regression showed that the EI, corrected EI, MT of MG, and corrected EI of TA might predict DPN (Table 3). Higher EI and corrected EI of MG, higher corrected EI of TA, and lower MT of MG might be prone to have DPN. To be noticed in our study, the clinical characteristics between the two groups had some contradictions compared with other reports that need to be explained. Unlike previous articles [1, 25], the levels of TG and TC were higher in the group without DPN, which might result from the lipid-lowering effects of antidiabetic drugs and lipid-lowering drugs for preventing the development of PAD. However, due to the change and combined use of different medicines, the influence of drugs was hard to eliminate. Besides, although diabetic kidney disease and diabetic retinopathy were the risk factors of DPN in other studies [4], the sample size and criteria excluding uremia decreased the effect.

The findings proved that muscle atrophy of proximal muscle groups of lower extremities occurred in the early DPN. According to our study, EI and corrected EI of MG and TA might be measurement indexes for supervising the progression of DPN. However, whether muscle ultrasound could be applied to assess exercise benefits for T2DM patients with DPN still needs further study.

## 5. Limitation

Firstly, as exploratory research, motor function assessment, nerve conduction, and other DPN diagnostic methods were not investigated in our study. And the influence of drugs was not under control. Secondly, the sample size was small, which might minimize the significance of some variates, like the CSA of RF. Finally, we did not discuss the difference between the dominance and nondominance of lower limbs. Although the differences between the dominant and nondominant sides have been reported in the literature, the relevant research results are controversial. Meanwhile, unlike upper limb dominance, the definition of lower limb dominance was unclear and it should be determined by strength testing [26].

## 6. Conclusion

Above all, the muscle parameters of MG (MT, EI, and corrected EI) and TA (EI) were associated with DPN, respectively, reflecting single muscle mass or muscle quality. Corrected EI or EI of MG combined with CSA of RF, PAD, and sex was associated with VPT significantly, which supported that muscle ultrasound might be a substantial quantitative tool for detecting the exercise benefits for DPN. Further studies about exercise focusing on MG and TA among T2DM patients with DPN were recommended.

## Figures and Tables

**Table 1:** Baseline characteristics of T2DM patients with and without DPN.

Characteristic	Non-DPN	DPN	*p* value
	*n* = 51	*n* = 39	
Sex, *n* (%)			0.887
Male	28 (54.9)	22 (56.4)	
Female	23 (45.1)	17 (43.6)	
Age (years, mean ± SD)	61.4 ± 9.92	64.8 ± 8.02	0.086
Weight (kg, mean ± SD)	63.3 ± 10.9	62.1 ± 11.9	0.615
Height (m, mean ± SD)^a^	1.63 ± 0.09	1.63 ± 0.07	0.993
BMI (kg/cm^2^, mean ± SD)	23.7 ± 3.28	23.3 ± 3.52	0.618
Smoking, *n* (%)	17 (33.3)	9 (23.1)	0.287
Alcohol, *n* (%)	7 (13.7)	4 (10.3)	0.619
Duration, *n* (%)			0.598
<10 years	22 (43.1)	19 (48.7)	
≥10 years	29 (56.9)	20 (51.3)	
Use of insulin (%)	14 (27.5)	9 (23.1)	0.637
Hypertension, *n* (%)	24 (47.1)	17 (43.6)	0.743
PAD of lower extremities, *n* (%)	24 (47.1)	28 (71.8)	0.019^∗^
DKD, *n* (%)			0.689
Stages 1-2	41 (80.4)	30 (76.9)	
Stages 3-4	10 (19.6)	9 (23.1)	
DR, *n* (%)	24 (47.1)	17 (43.6)	0.743
HbA1c (%)	9.97 ± 2.32	10.2 ± 2.50	0.633
TC (mmol/L, mean ± SD)	5.40 ± 1.59	4.82 ± 0.93	0.033^∗^
TG (mmol/L, median, Q1-Q3)^a^	1.58 (1.22-2.49)	1.26 (0.86-1.83)	0.020^∗^
HDL (mmol/L, mean ± SD)	1.17 ± 0.30	1.19 ± 0.31	0.729
LDL (mmol/L, mean ± SD)	3.13 ± 1.15	2.85 ± 0.73	0.182

^∗^ *p* < 0.05. ^a^Mann–Whitney *U* test. Other quantitative variates were analyzed by Student's *t*-test. Abbreviations: PAD: peripheral artery disease; DKD: diabetic kidney disease; DR: diabetic retinopathy; BMI: body mass index; TG: triglycerides; TC: total cholesterol; HDL: high-density lipoprotein; LDL: low-density lipoprotein; HbA1c: glycated hemoglobin.

**Table 2:** Relationship between muscle ultrasound parameters and DPN.

	Non-DPN	DPN	*p* value
	*n* = 51	*n* = 39	
Rectus femoris			
EI (UA, mean ± SD)	25.4 ± 7.48	28.8 ± 7.51	0.035^∗^
CSA (cm^2^, mean ± SD)	4.95 ± 1.06	4.56 ± 1.13	0.094
MT (cm, mean ± SD)	1.38 ± 0.23	1.28 ± 0.27	0.049^∗^
Corrected EI^a^ (UA, mean ± SD)	56.3 ± 17.5	58.5 ± 14.6	0.286
Tibialis anterior			
EI (UA, mean ± SD)	28.1 ± 7.29	32.3 ± 7.10	0.007^∗∗^
CSA^a^ (cm^2^, mean ± SD)	3.01 ± 0.97	2.72 ± 0.82	0.117
MT (cm, mean ± SD)	1.72 ± 0.28	1.63 ± 0.30	0.105
Corrected EI^a^ (UA, mean ± SD)	37.3 ± 12.0	40.9 ± 8.27	0.023^∗^
Medial head of gastrocnemius			
EI (UA, mean ± SD)	18.9 ± 6.01	23.5 ± 6.94	0.001^∗∗^
CSA (cm^2^, mean ± SD)	4.56 ± 1.10	4.20 ± 0.88	0.094
MT^a^ (cm, mean ± SD)	2.10 ± 0.26	1.96 ± 0.27	0.029^∗^
Corrected EI (UA, mean ± SD)	29.6 ± 7.27	34.3 ± 8.75	0.006^∗∗^

^∗^ *p* < 0.05 and ^∗∗^*p* < 0.01. ^a^Mann–Whitney *U* test. Other quantitative variates were analyzed by Student's *t*-test. Abbreviations: EI: echo intensity; CSA: cross-sectional area; MT: muscle thickness.

**Table 3:** Binary logistic regression results of different muscle parameters.

	OR (95% CI)	*p* value
Model 1		
Echo intensity of MG	1.114 (1.039, 1.196)	0.003^∗∗^
TG		0.360
TC		0.338
PAD		0.114
Model 2		
Echo intensity of TA	1.086 (1.020, 1.157)	0.010^∗^
TG		0.260
TC		0.134
PAD		0.056
Model 3		
Corrected echo intensity of MG	1.079 (1.019, 1.142)	0.009^∗∗^
TG		0.135
TC		0.125
PAD		0.072
Model 4		
Muscle thickness of MG	0.153 (0.025, 0.937)	0.042^∗^
PAD of lower extremities	2.640 (1.060, 6.575)	0.037^∗^
TG		0.250
TC		0.163

^∗^ *p* < 0.05, ^∗∗^*p* < 0.01, and ^∗∗∗^*p* < 0.001. Abbreviations: CI: confidence interval; OR: odds ratio; RF: rectus femoris; TA: tibialis anterior; MG: medial head of gastrocnemius; PAD: peripheral artery disease; TG: triglycerides; TC: total cholesterol.

**Table 4:** Correlation between VPT, muscle parameters, and clinical data.

	*r*	*p* value
Rectus femoris		
Echo intensity	0.219	0.003^∗∗^
Cross-sectional area	-0.224	0.002^∗∗^
Muscle thickness	-0.236	0.001^∗∗^
Corrected echo intensity	-0.051	0.496
Tibialis anterior		
Echo intensity	0.176	0.018^∗^
Cross-sectional area	-0.224	0.002^∗∗^
Muscle thickness	-0.236	0.001^∗∗^
Corrected echo intensity	0.105	0.162
Medial head of gastrocnemius		
Echo intensity	0.255	<0.001^∗∗∗^
Cross-sectional area	-0.179	0.017^∗^
Muscle thickness	-0.243	0.001^∗∗^
Corrected echo intensity	0.210	0.005^∗∗^
Other variates		
Age	0.281	<0.001^∗∗∗^
Weight	-0.042	0.573
Height	0.119	0.111
BMI	-0.105	0.161
HbA1c	0.085	0.255
TC	-0.124	0.096
TG	-0.218	0.003^∗∗^
HDL	-0.012	0.874
LDL	-0.053	0.485

^∗^ *p* < 0.05, ^∗∗^*p* < 0.01, and ^∗∗∗^*p* < 0.001. Abbreviations: BMI: body mass index; TG: triglycerides; TC: total cholesterol; HDL: high-density lipoprotein; LDL: low-density lipoprotein; HbA1c: glycated hemoglobin.

**Table 5:** Multiple linear regression of variables correlated with VPT.

	Nonstandard *β*	Standard *β*	95% CI	*p* value	*R*	*R* ^2^
Model 1					0.495	0.245
PAD	3.988	0.217	1.491~6.485	0.002		
CSA of RF	-2.197	-0.29	-3.290~-1.104	<0.001		
Sex	-5.198	-0.284	-7.890~-2.506	<0.001		
Corrected EI of MG	0.251	0.242	0.104~0.398	<0.001		
Model 2					0.488	0.238
PAD	3.952	0.215	1.425~6.480	0.002		
CSA of RF	-2.243	-0.296	-3.340~-1.147	<0.001		
Sex	-4.126	-0.225	-6.743~-1.509	0.002		
EI of MG	0.272	0.215	0.099~0.444	0.002		

Abbreviations: CI: confidence interval; RF: rectus femoris; MG: medial head of gastrocnemius; PAD: peripheral artery disease; EI: echo intensity; CSA: cross-sectional area.

## Data Availability

The data used to support the findings of this study are available from the corresponding author or the first author upon reasonable request.
